# The effect of caffeine on cisplatin-induced apoptosis of lung cancer cells

**DOI:** 10.1186/2162-3619-4-5

**Published:** 2015-02-11

**Authors:** Gan Wang, Vanitha Bhoopalan, David Wang, Le Wang, Xiaoxin Xu

**Affiliations:** Institute of Environmental Health Sciences, Wayne State University, 259 Mack Avenue, Detroit, MI 48201 USA

**Keywords:** Cisplatin treatment, Caffeine, ATR inhibition, ATM activation, PUMA expression, Apoptosis, Lung cancer cells

## Abstract

**Background:**

Cisplatin is an important DNA-damaging anticancer drug that has been used to treat many cancer types. However, the effectiveness of cisplatin treatment diminishes quickly as cancer cells develop resistance to the drug, which eventually results in treatment failure. Caffeine is an ingredient contained in many food sources. Caffeine can inhibit activities of both ATM and ATR, two important protein kinases involved in DNA damage-induced cell cycle arrest and apoptosis. The effect of caffeine on cisplatin-based cancer treatment is not well known.

**Methods:**

Caspase-3 activation and cell growth inhibition assays were used to determine the effect of caffeine on cisplatin-induced apoptosis and cell growth in lung cancer cells. Real time PCR, immunoblotting, and flow cytometry assays were used determine a mechanism through which the presence of caffeine increased cisplatin-induced apoptosis of the lung cancer cells.

**Results:**

Our caspase-3 activation studies demonstrated that the presence of caffeine increased the cisplatin-induced apoptosis in both HTB182 and CRL5985 lung cancer cells. Our cell growth inhibition studies indicated that the presence of caffeine caused a more increase for cisplatin-induced cell growth inhibition. The results obtained from our real time PCR and western blot studies revealed that the presence of caffeine increased cisplatin-induced expression of the PUMA pro-apoptotic protein in these lung cancer cells. The results of our protein phosphorylation studies indicated that the presence of caffeine caused a decrease in CHK1 phosphorylation at Ser^317^/Ser^345^ but an increase in ATM phosphorylation at Ser^1981^ in the lung cancer cells treated with cisplatin. In addition, our flow cytometry studies also revealed that the presence of caffeine caused an increase in G1 cell population but a decrease for cisplatin-induced cell cycle arrests at the S and the G2 checkpoints in HTB182 and CRL5985 cells respectively.

**Conclusion:**

Our results suggest that the presence of caffeine increases the cisplatin-induced lung cancer cell killings by inhibiting ATR but inducing ATM activation, resulting in an increase in expression of *PUMA* protein and an increase in apoptosis.

## Introduction

Cisplatin is an anticancer drug that has been used to treat many cancer types [1]. However, the effectiveness of this drug often diminishes quickly due to development of cancer cell resistance to this drug [2]. Therefore, approaches that can reverse cancer cell resistance phenotype or potentiate cancer cells to cisplatin treatment need to be explored.

The anticancer action of cisplatin results from its ability to generate DNA damage (*e.g.* intra- and inter-strand DNA crosslinks) and to promote DNA damage-induced cell cycle arrest and apoptosis [2, 3]. However, cancer cells can minimize or overcome the cytotoxicity of cisplatin using several different cellular mechanisms [2]. Nucleotide excision repair (NER), the major DNA repair pathway used to repair bulky DNA damage generated by many environmental carcinogens and therapeutic drugs, is one of the major mechanisms used to remove cisplatin DNA damage in cancer cells [4, 5]. In addition, cancer cells can also minimize the cytotoxicity of cisplatin with other cellular mechanisms, such as altering cell membrane permeability to reduce cellular uptake of cisplatin [1, 6]. If the signal of cisplatin-induced cell cycle arrest/apoptosis could be enhanced, the effectiveness of cisplatin in cancer treatment would be greatly improved.

Caffeine is an ingredient contained in many of our dietary sources, especially in beverages such as coffee, tea, and other soft drinks. Caffeine is a known central nervous system stimulant that acts through adenosine receptors and monoamine neurotransmitters [7]. Caffeine is also a protein kinase inhibitor that inhibits a variety of protein kinases [8], including ATM and ATR, two important protein kinases involved in DNA damage-induced cell cycle arrest and the apoptosis signaling process [5, 9, 10]. Caffeine consumption is known to lower cancer risk for certain types of cancer [11]. In addition, studies reveal that caffeine inhibits DNA repair [12, 13]. However, the effect of caffeine consumption on cisplatin-based cancer treatment is unknown.

We have studied the effect of caffeine on cisplatin-induced apoptosis of lung cancer cells. Using HTB182 lung squamous carcinoma cells and CRL5985 lung adenocarcinoma cells, the results of our caspase-3 activation studies demonstrated that the presence of caffeine significantly increased the cisplatin-induced caspase-3 activation in these lung cancer cells. The results of our cell survival studies revealed that the presence of caffeine enhanced cisplatin-induced cell growth inhibition and apoptosis in these lung cancer cells. The results of our real time PCR and western blotting studies indicated that the presence of caffeine increased cisplatin-induced expression of the *PUMA* pro-apoptotic protein in these lung cancer cells. The results of our protein phosphorylation studies further revealed that the presence of caffeine caused a decrease in CHK1 phosphorylation at Ser^317^/Ser^345^ but an increase in ATM phosphorylation at Ser^1981^ in the cisplatin-treated HTB182 and CRL5985 lung cancer cells. In addition, the results of our flow cytometry studies also demonstrated that the presence of caffeine caused an increase in G1 but a decrease in either S or G2 phase cell population for the cisplatin-treated HTB182 and CRL5985 lung cancer cells. All of these results suggest that the presence of caffeine increases the cisplatin-induced cell killing in both CRL5985 and HTB182 lung cancer cells by inhibiting ATR activity, resulting in an increase in apoptosis.

## Results

### The presence of caffeine caused a greater increase of cisplatin-induced caspase-3 activation in both HTB182 and CRL5985 lung cancer cells

**Figure 1:**
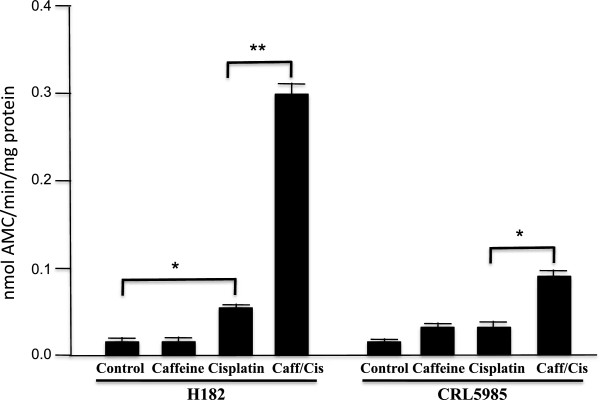
**Cisplatin-induced caspase-3 activation in both HTB182 and CRL5985 lung cancer cells.** The lung cancer cells were first treated with caffeine (2 mM) for 24 hours and then with cisplatin (10 μM) for 36 hours before caspase-3 activity was measured. The caspase-3 activity was determined using the Ac-DEVD-AMC as a substrate and measured as nmol AMC/min/mg protein. The *p* value <0.01 was considered statistically significant in this study (*p value < 0.01; **p value < 0.001).

To determine the effect of caffeine on cisplatin-based cancer treatment, we first determined the effect of caffeine on cisplatin-induced caspase-3 activation in both HTB182 and CRL5985 lung cancer cells. The HTB182 and CRL5985 lung cancer cells were treated either with cisplatin (10 μM) alone or in combination with both caffeine (2 mM) and cisplatin (10 μM) for 36 hours and caspase-3 activity was determined (Figure 1). As controls, caspase-3 activity was also determined from both untreated and caffeine-treated HTB182 and CRL5985 lung cancer cells (Figure 1). Very low caspase-3 activity was detected in the untreated or caffeine-treated HTB182 and CRL5985 cells (Figure 1). When treated by cisplatin alone, a small increase in caspase-3 activity was observed in both HTB182 and CRL5985 lung cancer cells (Figure 1). However, when treated with both caffeine and cisplatin, a significant increase in caspase-3 activity was observed in both HTB182 and CRL5985 lung cancer cells (Figure 1). These results suggest that the presence of caffeine increased the cisplatin-induced apoptosis in both HTB182 and CRL5985 lung cancer cells.

### The presence of caffeine resulted in an increase for cisplatin-mediated cell growth inhibition in both HTB182 and CRL5985 lung cancer cells

**Figure 2:**
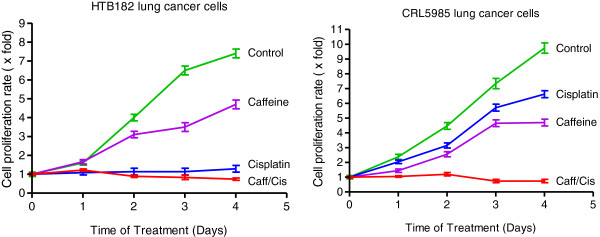
**The effect of caffeine on cisplatin-mediated cell growth inhibition in both HTB182 and CRL5985 lung cancer cells.** The cells were treated with 2 mM caffeine, 10 μM cisplatin, or a combination of 2 mM caffeine and 10 μM cisplatin. The cell growth rate was determined at 24, 48, 72, and 96 hours after the treatment.

To further determine the role of caffeine in cisplatin-based cancer treatment, we studied the effect of caffeine on cisplatin-mediated cell growth inhibition of HTB182 and CRL5985 lung cancer cells (Figure 2). When treated with caffeine alone, moderate inhibition in cell growth was observed in both HTB182 and CRL5985 lung cancer cells (Figure 2). When treated with cisplatin alone, some cell growth inhibition was observed in HTB182 cells but little effect on cell growth was observed in CRL5985 cells (Figure 2). However, when treated with the combination of caffeine and cisplatin, a much more severe inhibition in cell growth was observed in both HTB182 and CRL5985 lung cancer cells. In fact, the number of live cells was significantly more decreased for the cells treated with both caffeine and cisplatin than the untreated cells or the cells treated with cisplatin alone at three and four days for both HTB182 and CRL5985 cells (Figure 2). These results suggest that the presence of caffeine not only enhances the cisplatin-induced cell growth inhibition but also increases the cisplatin-induced apoptosis in both HTB182 and CRL5985 lung cancer cells.

### The presence of caffeine led to a greater increase of cisplatin-induced PUMA expression in both HTB182 and CRL5985 lung cancer cells

**Figure 3:**
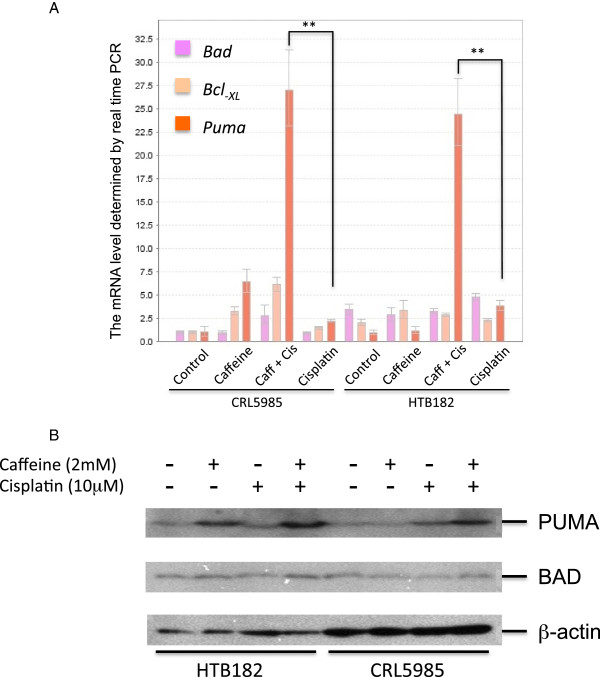
**Detection of Bad, Bcl**_**-XL**_ **, and** ***Puma*** **expressions in both untreated and caffeine/cisplatin-treated HTB182 and CRL5985 lung cancer cells. (A)** The levels of *Bad*, *Bcl*_*-XL*_, and *Puma* mRNAs in both untreated and caffeine/cisplatin-treated HTB182 and CRL5985 lung cancer cells as determined by real time PCR assay. **(B)** The levels of PUMA and BAD protein in both untreated and caffeine/cisplatin-treated HTB182 and CRL5985 lung cancer cells as determined by western blot assay. The *p* value <0.01 was considered statistically significant in this study (**p value < 0.001).

To define a mechanism through which the presence of caffeine caused an increase of cisplatin-induced apoptosis in both HTB182 and CRL5985 lung cancer cells, we determined the expression levels of BAD, Bcl_-XL_, and *PUMA*, three important proteins involved in the DNA damage-induced apoptosis process [14], in both untreated and cisplatin/caffeine-treated HTB182 and CRL5985 lung cancer cells (Figure 3). We first determined the mRNA levels of *Bad*, *Bcl*_*-XL*_, and *Puma* genes in both untreated and cisplatin/caffeine-treated HTB182 and CRL5985 cells using a reverse transcription-based mRNA quantitation (real time PCR) assay (Figure 3A). When treated with caffeine, some increase in mRNA levels was observed for both *Bcl*_*-XL*_ and *Puma* genes in the CRL5985 but not in the HTB182 lung cancer cells (Figure 3A). When treated with cisplatin, no significant increase was observed in any of the *Bad*, *Bcl*_*-XL*_, or *Puma* genes in either CRL5985 or HTB182 lung cancer cells (Figure 3A). When treated with both caffeine and cisplatin, however, a significantly greater increase in *Puma* mRNA was observed in both HTB182 and CRL5985 lung cancer cells (Figure 3A). Some increase in *Bcl*_*-XL*_ mRNA was also observed in the CRL5985 cells treated with both caffeine and cisplatin (Figure 3A). However, this increase was not statistically significant in comparison to the *Bcl*_*-XL*_ mRNA level in CRL5985 cells treated with caffeine alone (Figure 3A).

We further determined the protein levels of PUMA and BAD proteins in both untreated and caffeine/cisplatin-treated HTB182 and CRL5985 lung cancer cells (Figure 3B). The results of our immuno-blotting studies revealed that the cells treated with both caffeine and cisplatin displayed higher levels of PUMA protein than the cells treated by caffeine or cisplatin alone in both HTB182 and CRL5985 cells (Figure 3B). However, the protein level of the BAD, another pro-apoptotic protein, remained unchanged with or without the caffeine and/or cisplatin treatment in both HTB182 and CRL5985 cells (Figure 3B).

Given the important role of PUMA protein in DNA damage-induced apoptosis, these results suggest that induced expression of PUMA protein plays an important role in caffeine increasing cisplatin-induced apoptosis of HTB182 and CRL5985 lung cancer cells.

### The presence of caffeine caused a decrease of CHK1 phosphorylation at Ser^317^/Ser^345^ and an increase of ATM phosphorylation at Ser^1981^ in the cisplatin-treated HTB182 and CRL5985 lung cancer cells

**Figure 4:**
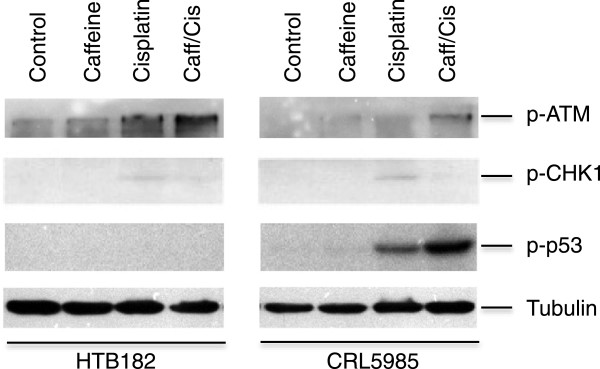
**The effect of caffeine on cisplatin treatment-induced ATM, CHK1, and p53 phosphorylation in both HTB182 and CRL5985 lung cancer cells.** The cells were treated with caffeine (2 mM) for 24 hours before the cisplatin treatment (10 μM). The cells were collected 20 hours after the cisplatin treatment and used for western blotting.

To define a mechanism through which the presence of caffeine caused an increase of cisplatin-induced apoptosis in the lung cancer cells, we determined the phosphorylation levels of CHK1 (Ser^317^/Ser^345^), ATM (Ser^1981^), and p53 (Ser^15^), three proteins involved in the DNA damage-induced signaling process (5,8), from both untreated and caffeine/cisplatin-treated HTB182 and CRL5985 lung cancer cells (Figure 4). The results of our western blotting revealed that the presence of caffeine caused a decrease of CHK1 phosphorylation at Ser^317^/Ser^345^ but an increase of ATM phosphorylation at Ser^1981^ in the cisplatin-treated HTB182 and CRL5985 lung cancer cells (Figure 4). Interestingly, the p53 phosphorylation at Ser^15^ was detected only in the cisplatin-treated CRL5985 cells and the presence of caffeine increased this cisplatin-induced p53 phosphorylation in the CRL5985 cells (Figure 4). Since the Ser^317^/Ser^345^ of CHK1 protein are phosphorylated primarily by ATR kinase [15, 16], these results suggest that the ATR activity was inhibited in these lung cancer cells.

### The presence of caffeine altered cell cycle profile of the cisplatin-treated HTB182 and CRL5895 lung cancer cells

**Figure 5:**
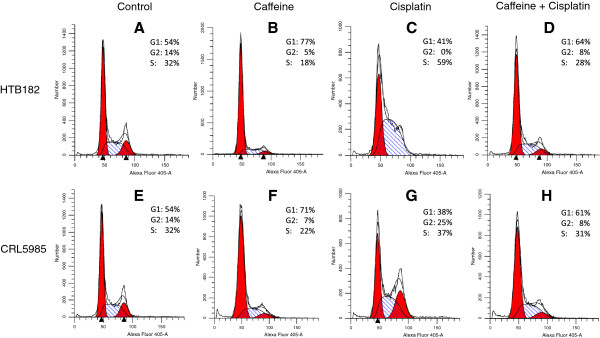
**The cell cycle profiles of both untreated and caffeine/cisplatin-treated HTB182 and CRL5985 lung cancer cells.** Cells were either untreated or treated with caffeine (2 mM) and/or cisplatin (10 μM). The cells were collected 20 hours after the cisplatin treatment and used for flow cytometry assay. A propidium iodide-based DNA staining was used in the flow cytometry assay to stain DNA content in the fixed cells. The corresponding captions used in the panels: **(A)** untreated HTB182; **(B)** HTB182 treated with caffeine; **(C)** HTB182 treated with cisplatin; **(D)** HTB182 treated with both caffeine and cisplatin; **(E)** untreated CRL5985; **(F)** CRL5985 treated with caffeine; **(G)** CRL5985 treated with cisplatin; **(H)** CRL5985 treated with both caffeine and cisplatin.

To further determine the mechanism through which the presence of caffeine caused an increase of cisplatin-induced apoptosis in the lung cancer cells, we also performed a flow cytometry assay to determine the cell cycle profiles of the HTB182 and CRL5985 lung cancer cells treated with caffeine and/or cisplatin (Figure 5). The presence of caffeine resulted in an accumulation of G1 cell population in both HTB182 and CRL5985 lung cancer cells (Figure 5B vs A and F vs E). The cisplatin treatment caused a S-phase arrest in the HTB182 cells (Figure 5C vs A) and a G2-phase arrest in the CRL5985 cells (Figure 5G vs E). When treated with both caffeine and cisplatin, however, the cisplatin-caused S and G2 arrests in these cells were diminished; instead, an increased G1-phase cell population was observed in these cells (Figure 5D vs 5C and 5H vs 5G). This result suggests that the presence of caffeine altered cells cycle profiles in the cisplatin-treated HTB182 and CRL5985 lung cancer cells.

## Discussion

In this study, we determined the effect of caffeine on cisplatin-induced apoptosis in lung cancer cells. The results of our caspase-3 activation studies demonstrated that the presence of caffeine caused a significant increase of cisplatin-induced caspase-3 activation compared to cells treated by cisplatin alone in both HTB182 and CRL5985 lung cancer cells. The results of our cell survival studies revealed that the presence of caffeine increased the cisplatin-induced cell growth inhibition and cell killing in these lung cancer cells. The results of our real time PCR and western blotting studies revealed that the presence of caffeine caused a significant increase of cisplatin-induced expression of PUMA protein in both HTB182 and CRL5985 lung cancer cells compared to cells treated by cisplatin alone*.* The results of our protein phosphorylation studies further demonstrated that the presence of caffeine caused a decrease in CHK1 phosphorylation at Ser^317^/Ser^345^ but an increase in ATM phosphorylation at Ser^1981^ in the cisplatin-treated HTB182 and CRL5985 cells. In addition, the results of our flow cytometry studies revealed that the presence of caffeine caused a decrease of cisplatin-caused S- and G2-phase cell cycle arrests but an increase in G1 cell population in the HTB182 and CRL5985 cells respectively. All of these results suggest that the presence of caffeine potentiates both HTB182 and CRL5985 lung cancer cells to cisplatin treatment by inhibiting ATR activity but inducing ATM activation, resulting in an increase in expression of the PUMA protein and an increase in apoptosis. These results not only determined the effect of caffeine on cisplatin-induced apoptosis in cancer treatment but also defined the molecular mechanism through which the presence of caffeine caused an increase of cisplatin-induced apoptosis in these lung cancer cells. Therefore, this information would have important implications in cancer treatment, especially in platinum-based cancer treatment.

Caffeine is a well-known protein kinase inhibitor that inhibits a variety of protein kinases, including ATM and ATR, two important protein kinases involved in DNA damage-induced cell cycle arrest and apoptosis [17]. Our CHK1 phosphorylation studies revealed that the presence of caffeine caused a decrease of cisplatin-induced CHK1 phosphorylation at Ser^317^/Ser^345^ in both HTB182 and CRL5985 lung cancer cells, suggesting an inhibited ATR activity in these cells [17, 18]. The mechanism through which inhibiting the ATR activity causes an increase of cisplatin-induced apoptosis in these lung cancer cells is unknown. However, work of others has demonstrate the essential role of ATR-CHK1 pathway in cell survival [19]. The results of our ATM phosphorylation studies also revealed that the presence of caffeine caused an increase of cisplatin-induced ATM phosphorylation at Ser^1981^ in these lung cancer cells. Given the important role of ATM phosphorylation at Ser^1981^ in activating the ATM protein and subsequent the ATM signaling pathway [5, 20], it is possible that inhibiting the ATR-CHK1 pathway with caffeine enhances the cisplatin-induced activation of the ATM signal pathway, which further induces expression of important pro-apoptotic proteins, including PUMA, to result in an increase in apoptosis of these lung cancer cells. Although caffeine inhibits both ATM and ATR kinases activities, some studies suggest that this inhibition is more effective *in vitro* than inside cells [21]. It is possible that the level of caffeine used in our studies (2 mM) only effectively inhibits the ATR and the ATM activity maintained in these cells. In addition, it is also possible that inhibiting the ATR-CHK1 pathway with caffeine leads to activation of other kinases, especially the DNA-dependent protein kinase (DNA-PK), in the cisplatin-treated cells which then induces expression of the apoptotic proteins and leads to increased apoptosis. DNA-PK can phosphorylate the p53 protein at Ser^15^[22, 23] and DNA-PK can be activated by DNA strand breaks and DNA fragments [24]. Therefore, it would be important to further determine the role of the ATM and DNA-PK kinases in how caffeine potentiates these lung cancer cells to cisplatin treatment in future studies.

Both our real time PCR and western blot results demonstrated that the presence of caffeine caused an increase of cisplatin-induced expression of the *PUMA* protein in both HTB182 and CRL5985 lung cancer cells. Given the important role of PUMA in apoptosis [14], it is likely that induced expression of the *PUMA* protein plays a role in caffeine potentiating lung cancer cells to cisplatin treatment. Works of others have demonstrated that both p53 and NF-κB can induce expression of the PUMA protein [25, 26]. Our p53 phosphorylation studies revealed an induced p53 phosphorylation at Ser^15^ in the cisplatin-treated CRL5985, suggesting a role of p53 activation in the cisplatin-induced PUMA expression of CRL5985 lung cancer cells (Figure 4). However, the increased PUMA expression in cisplatin-treated HTB182 cells likely resulted from other mechanisms, especially through activation of the NF-κB protein. It is known that the NF-κB protein is inhibited by an active ATR protein [27]. It is possible that inhibiting the ATR activity with caffeine eliminates this inhibiting effect, which results in activation of the NF-κB and leads to a NF-κB dependent PUMA expression in the p53-deficient HTB182 cells. This observation has some very important clinical implications in cancer treatment since a majority of cancer patients are known to carry mutated or impaired p53 proteins [6] and react poorly to the platinum-based anticancer treatment.

The mechanism through which the presence of caffeine causes a greater increase of ATM activation in the cisplatin-treated HTB182 and CRL5985 lung cancer cells is unknown. One possible mechanism is that inhibiting ATR activity with caffeine may prevent the cisplatin-caused cell cycle arrest [28, 29] and continued cell cycle progression in the presence of cisplatin DNA damage leads to an increase in DNA strand breaks and causes activation of the ATM protein. Our flow cytometry study provides some evidence to support this explanation since our results revealed that the presence of caffeine reduced the cisplatin-induced S and G2 arrests in HTB182 and CRL5985 lung cancer cells. It is possible that the inability to arrest cells at the S and G2 phases in the cisplatin-treated cells causes a continued cell cycle progression, which results in an increase in DNA strand breaks and an increase in ATM activation.

The results of our flow cytometry studies revealed that cisplatin treatment caused S and G2 cell cycle arrests in HTB182 and CRL5985 lung cancer cells respectively whereas the presence of caffeine abolished this cisplatin-caused cell cycle arrest and resulted in an increase of the G1 cell population in these cells. This result is in agreement with our CHK1 phosphorylation results which demonstrate a decreased CHK1 phosphorylation at Ser^317^/Ser^345^ in the cisplatin-treated HTB182 and CRL5985 lung cancer cells in the presence of caffeine. Given the essential role of CHK1 phosphorylation in leading to S and G2 cell cycle arrests [30], it is likely that inhibiting the CHK1 phosphorylation with caffeine would eliminate the cisplatin-caused S and G2 arrests, leading to accumulation of G1 cells. In addition, this result also provides some important insight into the mechanism through which the presence of caffeine potentiates these lung cancer cells to cisplatin treatment. It is known that most tumor cells are deficient in the G1 checkpoint and reliant on the S and G2 checkpoints to arrest cell cycle for DNA repair and cell survival [31–33]; inhibiting the S and G2 checkpoints, therefore, would prevent this process and enhance the cisplatin-induced apoptotic signaling process in these tumor cells. Furthermore, it is known that one or more of cell cycle checkpoint response components may be mutated or impaired in cancer cells, inhibiting the remaining checkpoints likely will have a greater deleterious effect on cancer cells than on normal cells with regard to DNA-damaging anticancer drugs, resulting in increased efficiency and specificity of these drugs in cancer cell killing.

Caffeine is widely consumed through our food sources, especially through beverages such as coffee, tea, and other soft drinks. Although many studies have demonstrated the beneficial effect of caffeine consumption in lowering cancer risk [11], the effect of caffeine in cancer treatment, especially in platinum-based cancer treatment, is unknown. The results of our studies demonstrate that the presence of caffeine potentiates lung cancer cells to cisplatin-induced apoptosis. In addition, results obtained from our recent DNA methylation studies suggest that the presence of caffeine may prevent cigarette smoke-induced DNA hypermethylation of important tumor suppressor genes [34]. Therefore, it is possible that caffeine consumption provides many beneficial effects, including its effect on cancer prevention and treatment.

## Materials and methods

### Cell lines and primers used in the studies

Both HTB182 and CRL5985 cells were purchased from the American Type Culture Collection (ATCC) (Rockville, MD). The HTB182 were lung squamous carcinoma cells derived from a Stage-2 lung cancer patients. The CRL5985 were lung adenocarcinoma cells derived from a Stage-4 lung cancer patient. Both HTB182 and CRL5985 cells were maintained in RPMI1640 medium supplemented with 10% fetal bovine serum (FBS) and cultured at 37°C with 5% CO_2_.

The primers used in this study were listed in the Table 1 and were synthesized by the Midland Certified Reagent Company (Midland, TX). The PUMA primers were designed to bind to the *Puma* gene coding sequence between exon 2 and 3 to amplify a 200 bp cDNA fragment. The BAD primers were designed to bind to the *Bad* gene between exon 2 and 3 to amplify an 180 bp cDNA fragment. The BCL_-XL_ primers were designed to bind to the coding sequence of *Bcl*_*-XL*_ gene to amplify a 122 bp cDNA fragment. The β-Actin primers were designed to bind to the coding sequence of β-*actin* gene (within exon 4) to amplify a 175 bp cDNA fragment.

**Table 1:** **Primers used in the real time PCR studies**

1. PUMA primers:	
*PUMA forward primer:*	5′-CTCGCTCTCGCTGGCGGAGCAG-3′
*PUMA reverse primer:*	5′-CGCTGCTGCTCTTGTCTC-3′
2. BAD primers:	
*BAD forward primer:*	5′-GACGCCAGTCACCAGCAGGAG-3′
*BAD reverse primer:*	5′-CGTCACTCATCCTCCGGAGCTC-3′
3. Bcl_-XL_ primers:	
*Bcl*_*-XL*_ *forward primer:*	5′-GGTGAGTCGGATCGCAGCTTG-3′
*Bcl*_*-XL*_ *forward primer:*	5′-CTCTCGGCTGCTGCATTGTTC-3′
3. β-Actin Primers:	
*β-Actin forward primer:*	5′GTACGTTGCTATCCAGGCTGTG3′
*β-Actin reverse primer:*	5′CATGAGGTAGTCAGTCAGGTC-3′

### Caffeine and cisplatin treatment

Both caffeine and cisplatin were purchased from Sigma-Aldrich Company (St. Louis, MO). Caffeine was prepared as 100 mM stock in distilled water, filtered through a 0.2 μm filter, and stored at −80°C. Cisplatin was freshly prepared as a 100 mM solution in DMSO and used immediately for each experiment.

Cells were seeded onto 100 mm cell culture dishes and cultured at 37°C overnight to reach approximately 30% confluence. Some dishes were treated with caffeine (2 mM) for 24 hours. The freshly prepared cisplatin solution (100 mM) was then added into the cell growth medium to a final concentration of 10 μM. For both caspase-3 activation and western blotting of PUMA and Bcl-_XL_ studies, cells were collected 36 hours after the cisplatin treatment. For western blotting of CHK1, ATM, and p53 phosphorylation studies, cells were collected 20 hours after the cisplatin treatment.

### Cell survival assay

Cells were seeded at 100 mm dishes and incubated at 37°C overnight to reach a density of approximately 20% confluence. Cells were counted from one dish in each cell line and were used as starting cell number for the study. The rest of the dishes were treated with caffeine (2 mM), cisplatin (10 μM), or a combination of both caffeine (2 mM) and cisplatin (10 μM). Cells were counted from one set of dishes each day for four days. Trypan blue was used to distinguish between live and dead cells and only live cells were counted for the study. The experiments were repeated four times and the data was used to establish the cell growth curve using a GraphPad software.

### Caspase-3 activation and western blot assay

The caspase-3 activation assay was done using a protocol described in our previous study [35]. The western blot assay was done using a protocol described in our previous studies with some modification [35]. Essentially, 20 μg of total protein from each cell lysate was used in the western blots. The p-ATM (Ser^1981^), p-CHK1 (Ser^317^), pCHK1 (Ser^345^), and p-p53 (Ser^15^) antibodies were purchased from Cell Signaling Inc. (Beverly, MA). The PUMA (G-3), BCL_-XL_, β-actin, and Tubulin antibodies were purchased from Santa Cruz Biotechnologies Inc. (Santa Cruz, CA).

### Statistical analysis

All data was expressed as Mean ± standard deviation (S.D.). Statistically significant differences were determined using a student t-test with 95% confidence interval (CI). The data was obtained from at least three independent experiments.
